# PPS: Energy-Aware Grid-Based Coverage Path Planning for UAVs Using Area Partitioning in the Presence of NFZs

**DOI:** 10.3390/s20133742

**Published:** 2020-07-03

**Authors:** Alia Ghaddar, Ahmad Merei, Enrico Natalizio

**Affiliations:** 1 Department of Computer Science, International University of Beirut, P.O. Box 14-6404 Beirut, Lebanon; 41430485@students.liu.edu.lb; 2 Université de Lorraine, CNRS, LORIA, F-54000 Nancy, France

**Keywords:** Unmanned Aerial Vehicles, coverage path planning, energy-aware trajectories, remote sensing, grid-based technique, cellular decomposition, area partitioning, non-flying zones

## Abstract

Area monitoring and surveillance are some of the main applications for Unmanned Aerial Vehicle (UAV) networks. The scientific problem that arises from this application concerns the way the area must be covered to fulfill the mission requirements. One of the main challenges is to determine the paths for the UAVs that optimize the usage of resources while minimizing the mission time. Different approaches rely on area partitioning strategies. Depending on the size and complexity of the area to monitor, it is possible to decompose it exactly or approximately. This paper proposes a partitioning method called Parallel Partitioning along a Side (PPS). In the proposed method, grid-mapping and grid-subdivision of the area, as well as area partitioning are performed to plan the UAVs path. An extra challenge, also tackled in this work, is the presence of non-flying zones (NFZs). These zones are areas that UAVs must not cover or pass over it. The proposal is extensively evaluated, in comparison with existing approaches, to show that it enables UAVs to plan paths with minimum energy consumption, number of turns and completion time while at the same time increases the quality of coverage.

## 1. Introduction

An Unmanned Aerial Vehicle (UAV) (or commonly known as a drone) is an aircraft without an onboard human pilot and an unmanned type of vehicle [1]. UAVs are indeed a part of an Unmanned Aerial System (UAS); consisting of an Aircraft, a ground-based operator and a network infrastructure that links between them [2]. UAV flight can function with varying levels of control: either remotely operated by a human operator or autonomously operated via onboard computers [3].

UAVs are classified as aerial robots that are used for several tasks in different application domains. They can be used for Aerial Photography [4], or as Pesticide sprinkler [5], Ambulance UAVs [6], for search and rescue operations [7], disaster management [8,9,10], filming Sport Event [11], infrastructure inspection [12], air pollution monitoring [13], traffic policing and emergency response [14], etc. In the mentioned applications, UAVs could coordinate to establish an effective coverage path planning (CPP). Among the quality measures that classify the efficiency of a path planning method is the energy consumption and the mission completion time. These two metrics depend mainly on two factors: the trajectory length and the number of turns.

The quality of coverage (QoC) is also another quality metric to evaluate a path planning technique. The area coverage is one of the main challenging missions. More challenges can be considered during an area coverage mission, such as avoiding obstacles in 3-Dimensional [15] or 2-Dimensional environment [16], tracking moving objects [17], data collection [18], avoiding non-flying zones [16,19,20], etc. Depending on how large and how complex the area is, an exact or approximate cellular decomposition of the area can be used to support the coverage path planning operations, and to generate efficient paths.

Different CPP approaches cover the areas using single or multiple UAVs [19,21]. The number of UAVs is based on the mission needs. In the presence of multiple UAVs, a safety margin must be taken into consideration to avoid collisions and ensure efficient collaboration between them. For collision-free missions, large areas are partitioned into subareas. Each UAV is allowed to cover one or more subareas. The area is divided by lines named borders that define the subarea’s margins.

This paper extends our previous work [22], by proposing a new path planning algorithm to avoid obstacles and non-flying zones (NFZs). In the previous work, the total path is composed of partial paths that are parallel to the longest side of the area (could tracks), to reduce the number of turns. The paths are planned in areas without obstacles and NFZs. In this work, the main challenge is to avoid NFZs, while minimizing the number of turns needed to go around the areas. The paper presents an approach to optimize the area coverage and the mission completion time with the assistance of a single UAV or a network of UAVs. The main contributions in this study are listed below:

- We propose a partitioning method of the area of interest in the presence of NFZs. This method has the advantage of dividing the area into equal subareas. The partition borders in our work are chosen to be on the grid-cell boundaries in order to reduce the percentage of uncovered areas. However, other methods in the literature drop all the cells that are located on the boundaries, which may result in dropping parts of the area to cover, and in reducing the quality of coverage.
- Each partition, or subarea, is mapped into a grid graph. The graph is reduced to a sub-graph in which all the edges are weighted. A filtering technique is implemented to remove insignificant edges and nodes for the favor of getting an optimal path. This technique identifies worthy edges, and refines the selection of the candidate turning points compared to the previous work.
- We improve the turning points selection mechanism, by implementing a new cost function for edges. The defined cost function takes into consideration the edge completion time as well as the coverage rate while moving from one edge to another.
- We present a path planning algorithm that can be applied to areas with exact or approximate cellular decomposition.

The remainder of this paper is organized as follows: Section 2 discusses state of art; Section 3 describes the problem of the work; Section 4 shows the grid and graph representations; Section 5 provides details about our parallel partitioning method; Section 6 presents the evaluation metrics and results; and Section 7 concludes the paper.

## 2. Related Works

Coverage path planning (CPP) for the UAV network is a crucial problem in many application domains. It can be done in an online or offline mode according to the mission challenges and goals. One of the significant concerns in CPP is to ensure a total coverage of the entire region of interest. Different approaches in the literature are based on the grid-map representation and area partitioning [23]. The reason behind the area partitioning is to cover the region of interest using a UAV network, especially if the area is large and complex [24,25]. Grid-based techniques perform cellular decomposition to the zone of interest by placing a grid overlay on top of the area to simplify the coverage [24]. The workspace is thus divided into cells. The cellular decomposition can be either exact [25] or approximate [16]. Furthermore, CPP algorithms should be aware of existing obstacles or non-flying zones (NFZs). These zones are territories over which the UAVs are not allowed to fly, such as, zones alongside air terminals, sensitive areas or unessential structures [24].

### 2.1. Grid-Mapping Representation

In grid-based path planning, the area of interest is represented by a grid, in which each grid-cell dimension fits within the UAV footprint. The grid-mapping of the area can be done using 4-neighbors [26] or 8-neighbors solution [27]. The grid could be composed of square cells, rectangular or hexagonal cells [24].

#### 2.1.1. Exact Cellular Decomposition

Exact cellular decomposition [24] could be applied to regular or irregular areas. The planning strategies are mainly classified into two categories: individual and cooperative strategies. The individual strategy approaches use the exact cellular decomposition to plan the paths using a single UAV. They apply back-and-forth and spiral methods on concave and convex areas [21,25,28,29].

The cooperative strategies are applied in large areas with the usage of a UAV network, composed of multiple UAVs to cover an area of interest. The work in [30] uses back-and-forth on a convex polygonal area using multiple UAVs. The area is divided into three sub-regions. The distance between every two consecutive tracks is fit to the UAV’s footprint. Each UAV plans its path and, if there is any conflict in the paths, then new paths are generated to avoid collisions and other mission problems. The work in [31] provides a path planning algorithm to cover large complex polygonal areas using fixed-wing UAV in the presence of NFZs. The NFZs are adjacent to the area of interest. The authors provide an algorithm to decompose the area in a multiple convex polygons form. The provided path planning algorithm plans the tracks using the traditional back-and-forth boustrophedon technique. A cost function is implemented to reduce the mission completion time in the presence of wind. Other works apply spiral technique while using heterogeneous UAVs [25,29].

#### 2.1.2. Approximate Cellular Decomposition

The approximate cellular decomposition divides the area into a group of regular cells. These cells have normally a square structure, yet they can be represented in a trigonal or hexagonal structure. In many cases, the paths are planned in an offline mode, assuming full information about the area of interest, including the positions of the obstacles and the NFZs [24]. Obstacles and NFZ cells are assigned a value ”−1” [19,20].

Authors in [16] propose an energy-aware coverage path planning algorithm using a grid-based technique. They aim to reduce the energy consumption while planning paths over irregular areas. Their work enhances the grid-based algorithm presented in [19]. They evaluate their algorithm by using a novel energy cost function [32].

The path planning in [19] is divided into two parts. The first part consists of dividing the area into sub-areas. Each UAV calculates the cost of the path in one of the sub-areas. It finds the cost of moving from a cell to another with the angles performed at each turn. In the second part, the wave-front algorithm is applied on each path to minimize the flight time, the number of turns and the number of visited cells. The UAVs fly at the same altitude to fix the resolution of the camera.

Similarly, the coverage mission in [20] undergoes two stages: the area partitioning and the path planning. The number of partitions depends on the number of UAVs used. The reason behind the partitioning is to minimize overlapping between the sub-areas which is considered to be null, such that the union of the sub-tasks covers the original task.The algorithm uses Bresenham’s line algorithm (BLA) [33] with a recursive flood-fill algorithm, which picks an empty cells and floods in four direction while there are empty cells, and each cell flooded is marked as occupied. If the sub-areas do not contain all the needed cells, the algorithm re-starts the division process.

In their work, the authors propose an algorithm that follows the wave-front technique for the path planning phase. Furthermore, they use the distance transform and Breadth-First Search (BFS) for an efficient planning. The authors mentioned that the best path should contain a minimum number of turns. It also has to avoid visiting previously covered cells to lower the completion time. For this purpose, they use the deep-limiting search (DLS) algorithm, that ensure coverage completeness without revisited nodes. The algorithm searches among the nearest neighboring cells one to be the next UAVs position. Then a back-tracking algorithm is applied to form a tree of the generated coverage paths. Finally, they choose the path that has the least number of turns. It should be noted that authors do not consider the computational time since they plan the path in an offline mode.

Authors in [34] partition the area of interest-based on a clustering method and graph method. This work aims to generate paths without collisions for a team of UAVs that will cover the area. The generated paths are approximately equal in terms of length.

The work in [22] proposes an algorithm to cover an area of interest without obstacles and non-flying zones using one rotary-wing UAV. The algorithm uses a grid-based technique with approximate cellular decomposition and works in an offline mode. Each grid cell fits within the UAVs footprint and the cells are set in the same direction of the UAVs motion. Each grid cell has a positive value that represents the percentage of the area in it. The values are obtained from the heat-map then mapped between 0 and 1 after rescaling. A subdivision for the grid cells occurs to plan the path and to identify the turning points. The center of each four grouped subcells is considered as a vertex. The UAV passes through the vertices that have non-zero value.

**Figure 1:**
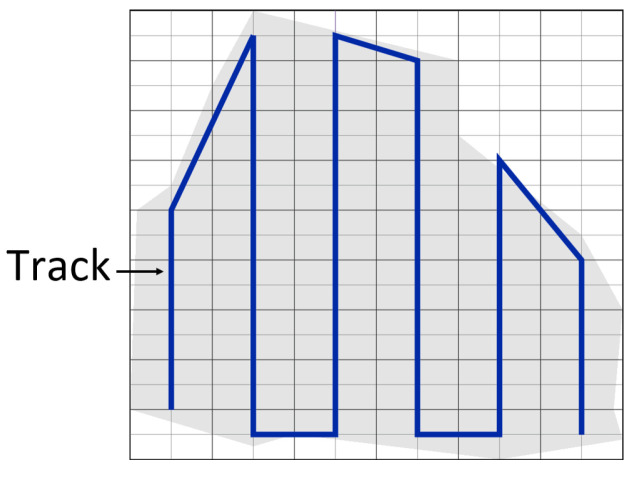
Coverage algorithm proposed in [22] for the area of interest.

It should be noted that the UAV moves in a direction parallel to the longest side of the grid as it helps to get a shorter path with the least number of turns. Figure 1 shows an example of an area of interest covered using our previous work [22], with a single UAVs. The algorithm generates an efficient path in terms of energy consumption and the completion time. It focuses on lowering the path length and number of turns. Different area shapes (regular and irregular) have been scanned in the absence of obstacles and non-flying zones. The drawback of this algorithm is that it does not identify the cells that include obstacles or non-flying zones from the other cells. Consequently, it cannot be applied to areas that include obstacles or non-flying zones.

In this paper, we aim to cover large areas in the presence of non-flying zones. We apply our partitioning method using scenarios with exact and approximate cellular decomposition using single and multiple UAVs. Below we present a short comparison between our work and some approaches in the literature works.

Table 1 summarizes the major features of the mentioned related works compared to our work. Mainly the entire area is divided using either approximate [16,19,20,34] or exact cellular [30] decomposition method, where our presented algorithm is applicable in both cases. Typically, some approaches use area decomposition techniques such as grid-based technique, that is used in the works of [16,19,20,34], wherein our work we use the grid-based technique with a subdivision that helps in planning accurate paths. Consequently, the grids are represented in graph form in some works as in [16,19,20,34]. In our work, we also convert the grid to a graph, but we provide a filtering method to easily manage the graph. Some approaches rely on weighted graphs by providing a cost function for the edges as in the works of [16,19,20], wherein our work we present an edge cost function that takes into account an extra parameter, which is coverage ratio for the edge. On the other hand, the coverage missions could be accomplished using either a single UAV [16] or a network of UAVs (multiple UAVs) [19,20,30,34]. In our work, we presented a path planning algorithm for single and multiple UAVs. Generally, the area of interest could include NFZs. These zones are either located inside [16,19,20,34] or around [16,34] the area of interest, wherein our work both NFZ locations are taken into account. While covering areas using a UAV network and/or avoiding NFZs, area partitioning algorithms are applied to divide the area [19,20,34]. The areas are divided using partition borders, in some cases border cells are dropped as in [19,20] which reduces the quality of coverage, especially if the cells include part of the area to be covered. In our work, we provide a partitioning algorithm that divides the area without omitting such needed cells. Finally, the performance of the path planning is evaluated using metrics such as energy consumption [16,34], completion time [16,19,20,30,34] and quality of coverage [19,20], where we use the three metrics to evaluate the efficiency of our planned paths.

## 3. Problem Formulation

In this work, we aim at efficiently covering an area of interest in the presence of non-flying zones using the partitioning method. One of the main challenges is to partition the area of interest into subareas that must be covered using a single or multiple UAVs. The requirements to fulfill for the path planning are:

- Avoid passing over the NFZs;
- Achieve full coverage of the area of interest;
- Ensure minimum energy consumption, by lowering the number of turns and the completion time.

The main steps of our work are as follows: (1) representing the area as a grid of cells (grid-division) and determining NFZ location (graph representation), (2) partitioning the area around the non-flying zone, (3) planning the path in each sub-area partition. We evaluate the performance of our partitioning method using scenarios with exact and approximate cellular decomposition using single and multiple UAVs.

**Figure 2:**
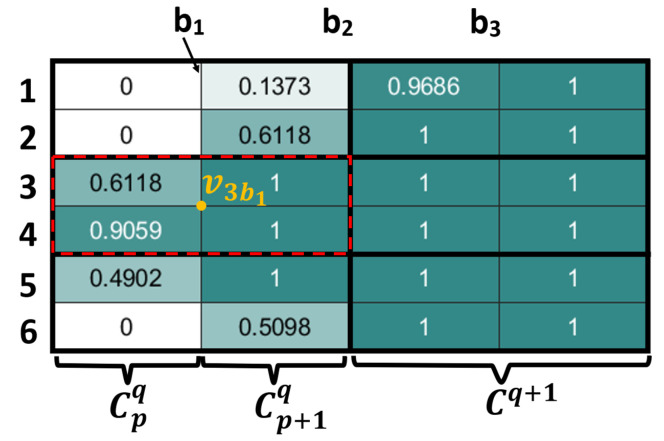
Mapping of the area of interest in a grid.

The investigated geographical area is denoted A. Similarly to [22], A is discretized into a grid. Each grid-cell has a rectangular shape and is divided into 4-adjacent sub-cells. Each cell has a positive value representing the percentage of area in it. A group of 4-adjacent sub-cells that includes three or four non-zero valued cells has a center *v*. We suppose that the UAV passes through the centers of the main grid-cells. We assume the grid is composed of n rows and m columns. The columns are denoted ${\left\{b_{k}\right\}}_{k=1}^{m}$. A center $v_{ib_{j}}$ is described by a pair of coordinates ($x_{i}$,$y_{i}$) and is located at row i and columns $b_{j}$ (see Figure 2).

A is partitioned into a set of *l* sub-areas, where $A=\cup {\left\{A_{k}\right\}}_{k=1}^{l}$. For every $A_{k}$, a graph $G_{k}$ is formed. The set of graphs is disconnected. We denote U the set of UAVs where ∣U∣≥ 1. We assume that the UAVs can communicate with each other, by using a WiFi or mobile cellular technology, and coordinate on the information to exploit to plan their paths collaboratively. For each UAV, we assign a path P. Each path is formed of a set of vertices and edges and has a set of turning points T with turning angles Φ. In the scenario where we use a single UAV, the total path $P_{total}=\cup {\left\{P_{k}\right\}}_{k=1}^{l}$. The aim is to find the shortest path that links all the partitions, avoids the NFZ and ensures full coverage with low energy consumption.

Formally, the grid is represented as a graph G(V,E,ζ), where V is the set of vertices (called also nodes), E⊆VXV is the set of edges, formed by pairs of vertices and ζ is the set of colors. Each edge is labeled with tuple of numeric values. The labeling of the edges is a mapping β:E→T x *C* where T=R and C={0,1}. Similarly, labeling of the vertices is a mapping α:V→ζ x K, where ζ is a set of colors {brown,black,blue,yellow,purple} and K={00,11,12,21,22} represents the set of keys. We will come back to the edge labels and keys in the next sections. We first explain how the grid is mapped and how the weighted graph is created. Then, we move to explain how the algorithm works. Table 2 defines the notation of relevant sets, indices, parameters and variables of our work.

## 4. Algorithm Design

**Figure 3:**
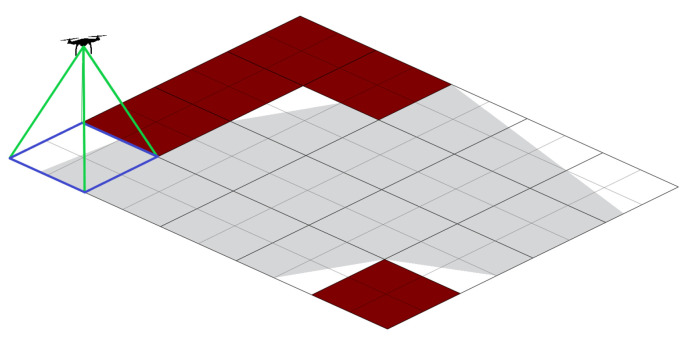
Unmanned Aerial Vehicle (UAV) footprint representation. Red boxes are non-flying zones (NFZ).

The grid-based technique decomposes the area of interest into cells. Grid cell size is determined by the resolution and field of view (FoV) for the camera called UAV footprint. Figure 3 shows a schematic of the field of view where the blue rectangle represents the UAV footprint. The area of interest is shown in grey color and red boxes represent NFZ.

**Figure 4:**
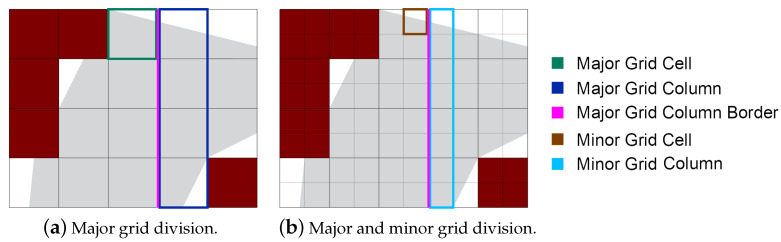
Grid-based area decomposition before and after subdivision.

Each grid cell has length *L* and width *W*. Concerning the scan direction of the UAV, the shortest side of the cell will be parallel to the longest side of the grid. If the UAV moves in a vertical track, then the shortest side of the cell will be parallel to the long side of the grid, and vice versa for the horizontal tracks. We apply a sub-division on the area to make efficient path planning to identify the non-zero value portions of the grid. This sub-division splits each major grid cell into four quarter sections called subcells (minor grid cells), as shown in Figure 4. Each group of four minor grid cells forms a UAV footprint, which represents the area that can be covered at once.

**Figure 5:**
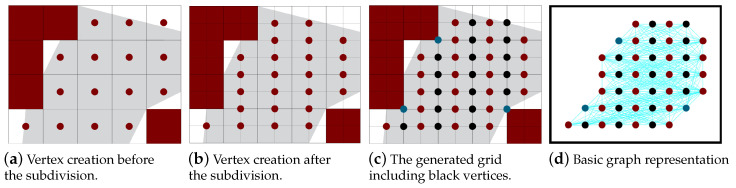
Vertex creation (before and after the sub-division) and the basic graph representation of the area.

Usually, the grid cells are represented by vertices at the center of each cell. Only non-zero cells are represented. These vertices are connected using the path planning algorithm. Figure 5a,b show the representation of the vertices before and after the sub-division. In the major grids, the vertices could represent semi-empty cells, while in the minor grids minor empty cells are not represented. The path tracks move through the columns in the major grid, and a turn occurs between them passing through their border. For better selection for the turning position, new helping vertices are represented on the border column as shown in Figure 5c (black vertices). The vertices are colored then the edges are created and assigned weights. Later the path is planned. Figure 5d represents the basic graph with all possible edges. In the below section, we present the different algorithms for vertex-coloring, edge creation and weighting and path planning.

### 4.1. Vertex-Coloring and Key-Assignment

The grid mapping process assigns each vertex a color and a key. The colors separate the main track vertices (i.e., brown nodes) from the helping vertices between two tracks (i.e., black nodes). Colors also differentiate border vertices (i.e., purple nodes) between the sub-areas. Moreover, vertex coloring helps in identifying special parts of the area such as NFZ borders (blue/yellow nodes). The brown nodes represent the vertices on the major grid column ($b_{j}$ where j is odd). Black ones represent the vertices on the major column border ($b_{j}$ where j is even). If a black vertex is located on the border of a non-flying zone then it will be represented by the blue color. These blue vertices provide useful information for the edge-creation algorithm, as they highlight the presence of a NFZ. In this way, the algorithm will not create an edge in this cell, to avoid the NFZ.

**Figure 6:**
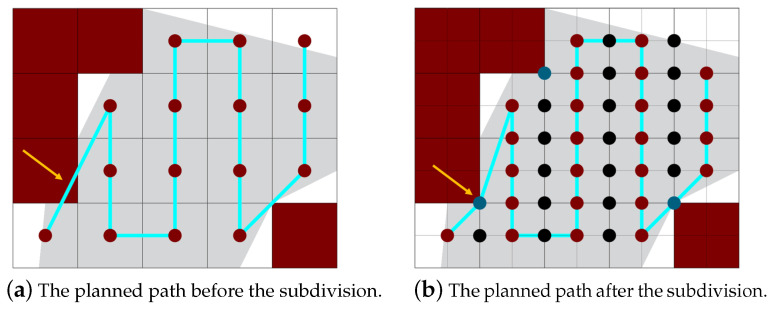
The difference between paths planned before and after the subdivision.

**Figure 7:**
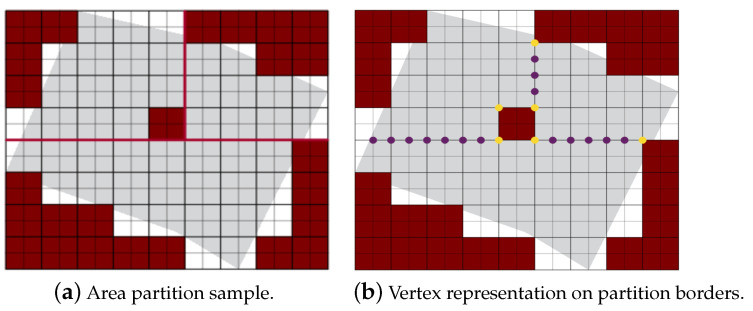
Partition borders vertex coloring.

Figure 6 shows the effect of considering blue vertices. In Figure 6a, the path is planned without the blue vertices. As you can see the path moves over an NFZ. On the contrary, in Figure 6b, with the help of the blue vertex, the planned path avoids the NFZ. On the other hand, in order to define the partition borders, a new set of vertices is created. The partition borders are represented by vertices with purple color, but if a vertex lies on an NFZ border, it changes the vertex color to yellow as shown in Figure 7. These vertices help in avoiding the collisions between different paths. They also help in finding the best joining edges between the paths in case a single UAV is used to cover the whole area. This mechanism is discussed in Section 5.3.

**Figure 8:**
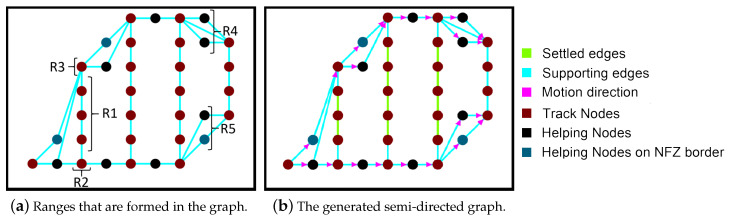
Edges creation: connecting vertices according to the rules.

The vertices are grouped into ranges to simplify the edge creation mechanism. This is an essential phase for the graph filtering. Ranges impose specific permissions on the vertices to form edges. The vertices are grouped into five ranges {R1, R2, R3, R4, R5} (see Figure 8a). Range 1 includes vertices that are not eligible to be turning points. Ranges 2 and 3 include brown candidate turning points. Ranges 4 and 5 include black candidate turning points. The ranges force the vertices to connect to certain neighboring nodes for better graph filtering. The edge creation is discussed in Section 4.2. Since there are two turning sides on a track we assign a key-value for each vertex in a range. The keys help in the path planning phase, where the algorithm searches for the best turning path between two tracks. This part will be discussed in Section 4.4.

Algorithm 1 assigns keys to brown colored vertices according to their location on the grid. Vertices that correspond to the first or last cells in the grid’s columns are more likely to be turning points (lines 1–4). Moreover, the vertices are divided into ranges to locate their positions that will help in then key assigning keys for each vertex (lines 5–30). A key value is assigned for each vertex in a range. The values of the keys are {00, 11, 12, 21, 22}. The vertices that belong to range 1 have a key equal to 00 (lines 31–33) and are not eligible to be turning points. The vertices that belong to range 2 and range 3 have a key equal to 12 or 11 (lines 34–39) and are considered candidate turning points. Algorithm 2 assigns keys to black colored vertices. Black vertices are divided into ranges according to their location with respect to the neighboring brown vertices in ranges 2 and 3 (lines 1–7). The vertices that belong to the range 4 and 5 will hold the key 21 and 22, respectively (lines 8–10, 11–13). In the next section, we will explain how the vertices are connected to form edges.
**Algorithm 1** Brown nodes key assignment**Input:**$C_{p}^{q}\wedge C_{p+1}^{q}\wedge b_{j}$  1: $F_{1}\leftarrow$ row of first vertex in $C_{p}^{q}$      2: $F_{2}\leftarrow$ row of first vertex in $C_{p+1}^{q}$    3: $L_{1}\leftarrow$ row of last vertex in $C_{p}^{q}$  4: $L_{2}\leftarrow$ row of last vertex in $C_{p+1}^{q}$    5: **for** (each $b_{j}$ where j is odd) **do**    6:     **if** $\left(F_{1}==F_{2}\right)$ **then**  7:         $R_{3}^{j}$=[$F_{1}:F_{2}$]  8:         $R_{1}^{j}.min$ =$F_{1}$+1  9:     **end if**  10:     **if**$\left(F_{1}<F_{2}\right)$**then**  11:        $R_{3}^{j}$=[$F_{2}+1:F_{1}$]  12:        $R_{1}^{j}.min$=$F_{2}$+2  13:    **end if**  14:    **if**$\left(F_{1}>F_{2}\right)$**then**  15:        $R_{3}^{j}$=[$F_{1}+1:F_{2}$]  16:        $R_{1}^{j}.min$=$F_{1}$+2  17:    **end if**  18:    **if**$\left(L_{1}==L_{2}\right)$**then**  19:        $R_{1}^{j}$=[$R_{1}^{j}.min$:$L_{1}-1$]  20:        $R_{2}^{j}$=[$L_{1}:L_{2}$]  21:    **end if**  22:    **if**$\left(L_{1}<L_{2}\right)$**then**  23:        $R_{1}^{j}$=[$R_{1}^{j}.min$:$L_{1}-2$]  24:        $R_{2}^{j}$=[$L_{1}-1:L_{2}$]  25:    **end if**  26:    **if**$\left(L_{1}>L_{2}\right)$**then**  27:        $R_{1}^{j}$=[$R_{1}^{j}.min$:$L_{2}-2$]  28:        $R_{2}^{j}$=[$L_{2}-2:L_{1}$]  29:    **end if**  30:**end for**  31:**for** (each $v_{ib_{j}}$ where i ∈$R_{1}^{j}$) **do**  32:    $v_{ib_{j}}$.setKey(00)  33:**end for**  34:**for** (each $v_{ib_{j}}$ where i ∈$R_{2}^{j}$) **do**  35:    $v_{ib_{j}}$.setKey(12)  36:**end for**  37:**for** (each $v_{ib_{j}}$ where i ∈$R_{3}^{j}$) **do**  38:    $v_{ib_{j}}$.setKey(11)  39:**end for**

**Algorithm 2** Black nodes key assignment

**Input:** $b_{j}$
  1: **if** (j is even) **then**  2:     $R_{4}^{j}.min$ = min{$R_{3}^{j-1}.min$,$R_{3}^{j+1}.min$}  3:     $R_{4}^{j}.max$= max{$R_{3}^{j-1}.max$, $R_{3}^{j+1}.max$}  4:     $R_{4}^{j}$ =[$R_{4}^{j}.min$,$R_{4}^{j}.max$]  5:     $R_{5}^{j}.min$= min{$R_{2}^{j-1}.min$,$R_{2}^{j+1}.min$}  6:     $R_{5}^{j}.max$ = max{$R_{2}^{j-1}.max$, $R_{2}^{j+1}.max$}  7:     $R_{5}^{j}$ =[$R_{5}^{j}.min$, $R_{5}^{j}.max$]

  8:     **for** (each $v_{ib_{j}}$ where i ∈$R_{4}^{j}$) **do**  9:         $v_{ib_{j}}$.setKey(21)  10:     
**end for**
  11:     **for** (each $v_{ib_{j}}$ where i ∈$R_{5}^{j}$) **do**  12:         $v_{ib_{j}}$.setKey(22)  13:     
**end for**
  14:
**end if**



### 4.2. Edge-Creation

An edge is a line joining a pair of vertices (called nodes in the graph). Figure 5d shows the basic graph representation of a grid. In our work, the edge creation abides by certain rules to filter the graph. The first rule is as follows, brown nodes in $b_{j}$ that fall in the range 1 can only form an edge with brown neighbors in the same range or with brown neighbors in $b_{j}$ that belong to range 2 and range 3. By this rule, we will be able to shape the main tracks. The second rule imposes the brown nodes in range 2 and (respectively range 3) to form edges with black nodes in $b_{j+1}$ in range 5 (respectively range 4). Moreover, brown nodes in range 2 (respectively range 3) in $b_{j}$ connect edges with the brown nodes in $b_{j+2}$ in range 2 (respectively range 3). In that way, the possible paths between the two main tracks are created. In the third rule, an edge could not be formed between two black nodes, additionally, black nodes that do not belong to range 4 or range 5 are omitted from the graph to avoid the formation of unneeded edges. Finally in the fourth rule, If a blue node falls at the beginning of a column $b_{j}$ (respectively at the end of column $b_{j}$), then all edges above (respectively below) the blue nodes will be removed to avoid NFZ.

Algorithm 3 joins vertices to form edges. Nodes that belong to range 3 in $b_{j}$ and $b_{j+2}$ are connected together (lines 1–5), then they are connected to the helping nodes in range 4 (lines 6–11). Similarly, the nodes that belong to range 2 in $b_{j}$ and $b_{j+2}$ are connected (lines 12–16). They also form edges with the helping nodes in range 5 (lines 17–22).
**Algorithm 3** Edge assignment to the nodes**Input:**$b_{j}$**Output:** edges  1: **for** (each $v_{ib_{j}}$ where i ∈$R_{3}^{j}$) **do**  2:    **for** (each $v_{ab_{j+2}}$ where a ∈$R_{3}^{j+2}$) **do**  3:         $v_{ib_{j}}$.setEdge($v_{ab_{j+2}}$)  4:     **end for**  5: **end for**  6: **for** (each $v_{ib_{j}}$ & $v_{ab_{j+2}}$ where i ∈$R_{3}^{j}$ and a ∈$R_{3}^{j+2}$) **do**  7:     **for** (each $v_{nb_{j+1}}$ where n ∈$R_{4}^{j+1}$) **do**  8:         $v_{ib_{j}}$.setEdge($v_{nb_{j+1}}$)  9:         $v_{ab_{j+2}}$.setEdge($v_{nb_{j+1}}$)  10:     **end for**  11:**end for**  12:**for** (each $v_{ib_{j}}$ where i ∈$R_{2}^{j}$) **do**  13:     **for** (each $v_{ab_{j+2}}$ where a ∈$R_{2}^{j+2}$) **do**  14:         $v_{ib_{j}}$.setEdge($v_{ab_{j+2}}$)  15:     **end for**  16:**end for**  17:**for** (each $v_{ib_{j}}$ & $v_{ab_{j+2}}$ where i ∈$R_{2}^{j}$ and a ∈$R_{2}^{j+2}$) **do**  18:     **for** (each $v_{nb_{j+1}}$ where n ∈$R_{5}^{j+1}$) **do**  19:         $v_{ib_{j}}$.setEdge($v_{nb_{j+1}}$)  20:         $v_{ab_{j+2}}$.setEdge($v_{nb_{j+1}}$)  21:     **end for**  22:**end for**

The generated graph has some edges with the direction associated with them. We call it a semi-directed graph in the sense that the movement direction of the UAV is not defined until the path planning step terminates. Figure 8b shows an example of the obtained semi-directed graph. Similarly to [22], the start point in our work is known. It is either the first or last brown point on the left column of the grid. If there is only one possible starting point, two keys could be assigned to this node (11 or 12).Consequently, two paths are generated, one path for each starting point. More details about this phase will be discussed in Section 4.4.

### 4.3. Edge-Weighting

Given two nodes *u*, v∈V, Let deg(v) be the degree (or valency) of a vertex *v* of a graph. It is the number of edges that are incident to *v*. Let $deg^{-}\left(v\right)$ be the indegree of vertex *v* in the graph and $deg^{+}\left(v\right)$ be the outdegree of *v*.

Every edge (u,v) is labeled with a tuple $\left(t_{uv},c_{uv}\right)$, where $t_{uv}$ represents the time needed for a UAV to move from vertex *u* to vertex *v*. The value $c_{uv}$ indicates whether the passage over the current edge will cover the nearby nodes (above or below *u* and *v*) according the turning node position. If so, the cost of the edge is 1 (i.e., $c_{uv}=1$), otherwise it is 0 (i.e., $c_{uv}=0$).

We denote by *D* the distance between two nodes *u* and *v* (Equation (1)).

(1) $$D_{\left(u,v\right)}=\sqrt{{\left(x_{u}-x_{v}\right)}^{2}+{\left(y_{u}-y_{v}\right)}^{2}}\;$$

Equation (2) calculates the exterior angle of the turn between two consecutive edges denoted $e_{1}=\left(v_{1},v^{{}^{\prime }}\right)$ and $e_{2}=\left(v^{{}^{\prime }},v_{2}\right)$. The angle is denoted $\Phi_{\hat{e_{1}e_{2}}}$:

(2) $$\Phi_{\hat{e_{1}e_{2}}}=\;\Phi_{\hat{v_{1}v^{{}^{\prime }}v_{2}}}=\pi -{cos}^{-1}\left[\frac{D_{\left(v_{1},\;v^{{}^{\prime }}\right)}^{2}+D_{\left(v^{{}^{\prime }},\;v_{2}\right)}^{2}-D_{\left(v_{1},\;v_{2}\right)}^{2}}{2D_{\left(v_{1},\;v^{{}^{\prime }}\right)}D_{\left(v^{{}^{\prime }},\;v_{2}\right)}}\right]\;\;$$

We denote $t_{uv}$ the time spent from node *u* to node *v*. It takes into consideration the cost of the angle performed while passing from a previous edge($v^{{}^{\prime }}u$) to the current edge(uv). It is defined as follows:

$$t_{uv}=Time\left(u,v\right)=\left\{\begin{array}{cc} 1\frac{D_{\left(u,v\right)}}{S} & deg^{-}\left(u\right)=0,deg^{+}\left(u\right)>=1\\ \frac{D_{\left(u,v\right)}}{S}+\frac{\Phi_{\hat{v^{{}^{\prime }}uv}}}{\omega } & deg^{-}\left(u\right)=1,v^{{}^{\prime }}\in L\left(u\right)\\ -1 & \mathrm{Otherwise} \end{array}\right.$$

(3) $$\;Time=\frac{D_{\left(V_{1},V_{2}\right)}}{S}+\frac{\Phi_{\hat{e_{1}e_{2}}}}{\omega }$$

where S and ω are parameters related to the UAVs speed and UAV rotation rate, we assume the UAV has constant speed. During the path planning, the edges having $t_{uv}=-1$, will have their cost modified according to the built path. The value of coverage *c* for an edge (u,v) is calculated as follows

- If the edge is between two brown nodes *u* and *v* and $u_{x}=v_{x}$, then $c_{uv}=1$.
- If the edge is between two turning points, check if the UAV footprint will be able to cover the surrounding neighboring nodes if it passes over this edge. For this purpose, Equation (4) is used. It represents the border line (denoted d1) of the footprint at that edge.

The equation of the line d1 is denoted f(u,v,x):

(4) f(u,v,x):y=ax+b

where the slope $a=\frac{y_{v}-y_{u}}{x_{v}-x_{u}}$ and

$$b=\left\{\begin{array}{cc} y_{u}-a*x_{u}+L/2 & \mathrm{key}(u)=11||\mathrm{key}(u)=21\\ y_{u}-a*x_{u}-L/2 & \mathrm{key}(u)=12||\mathrm{key}(u)=22\\ y_{u}-a*x_{u} & \mathrm{Otherwise} \end{array}\right.$$

If a vertex of the neighboring nodes to *u* and *v* (denoted $v^{{}^{\prime }}$), has key(u)={11,21} (respectively key(u)={12,22}), and falls above (respectively below) the line f(u,v,x), then $c_{uv}=0$, otherwise $c_{uv}=1$.

**Figure 9:**
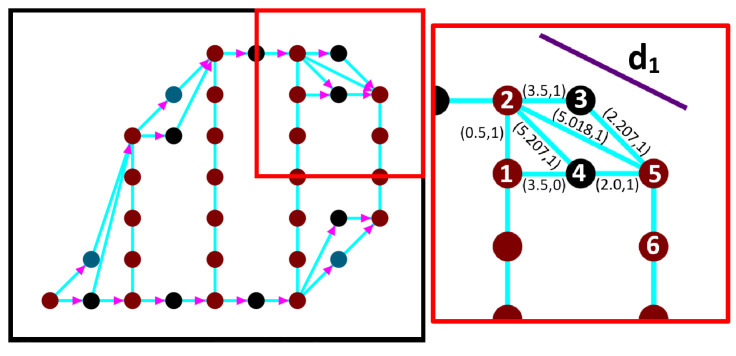
UAV coverage area while passing through edge (5,6).

Figure 9 shows a sample of edge weighting. Suppose the UAV passes from brown node 1 to the next brown node 5, with a speed of 10 unit/sec and a rotation rate 30 degree/sec. To find the coverage value for the edge $c_{25}$, we need to draw the line d1, which corresponds to the UAV footprint border while passing along the edge. In this case, we have the black node 3 above the edge $c_{25}$, we will calculate if black node 3 belongs to the line d1 or falls below the line, which means that it is covered.

### 4.4. Path Generation

Our aim is to find the path that covers the whole area with minimum energy consumption and completion time. Let us first present few notations: Let u⇝v denotes that *v* is a successor of *u*, and L(u) denotes the set of all predecessors of *u* that belong to G: $L\left(u\right)=\left\{v\in V_{G}v⇝u\right\}$. Let $u{⇝}^{*}v$ be the set of possible simple paths $\rho_{i}$ between *u* and *v*. For instance, if only two possible paths $\rho_{1}$ and $\rho_{2}$ exist, we say $u{⇝}^{*}v=\{u{⇝}_{\rho_{1}}v$, $u{⇝}_{\rho_{2}}v\}$. Let $V(u{⇝}_{\rho_{i}}v)$ be the set of vertices along the path $\rho_{i}$ between *u* and *v*.

(5) $$V\left(u{⇝}_{\rho_{i}}v\right)=\left\{v^{{}^{\prime }}\in Vu{⇝}^{*}v^{{}^{\prime }},v^{{}^{\prime }}{⇝}^{*}v\right\}\cup \left\{u,v\right\}$$

The set of edges along a path $\rho_{i}$ is denoted $E(u{⇝}_{\rho_{i}}v)$:

(6) $$E\left(u{⇝}_{\rho_{i}}v\right)=\left\{\left(a,b\right)\in Ea,b\in V\left(u{⇝}_{\rho_{i}}v\right)\right\}$$

The direct predecessor of vertex v along the path $\rho_{i}$ is denoted $L_{\rho_{i}}\left(v\right)$:

(7) $$L_{\rho_{i}}\left(v\right)=\left\{v^{{}^{\prime }}\in V\left(v^{{}^{\prime }},v\right)\in E\left(u{⇝}_{\rho_{i}}v\right)\right\}$$

The direct successor to vertex u along the path $\rho_{i}$ is denoted $L_{\rho_{i}}^{s}\left(u\right)$:

(8) $$L_{\rho_{i}}^{s}\left(u\right)=\left\{v^{{}^{\prime }}\in V\left(u,v^{{}^{\prime }}\right)\in E\left(u{⇝}_{\rho_{i}}v\right)\right\}$$

We denote $\varrho (u{⇝}^{*}v)$ all the possible paths between *u* and *v* such that all edges have c=1:

(9) $$\varrho \left(u{⇝}^{*}v\right)=\left\{\rho_{i}\in u{⇝}^{*}v\forall \left(a,b\right)\in E\left(u{⇝}_{\rho_{i}}v\right),c_{ab}=1\right\}$$

The goal is to find the list of connected edges (denoted χ) that form the shortest path in terms of time. The path should cover the whole area of interest. The main steps for the path generation in Algorithm 4 are as follows:

- Let I={V} be the list of vertices. Select the starting point.
- For each brown node $v_{ij}$, find the first brown successor $v_{pq}$ with which it has the lowest time cost segment. The vertex $v_{pq}\in I$ such that key$\left(v_{pq}\right)=\left\{00,11,12\right\}$ and $v_{ij}{⇝}^{*}v_{pq}$.
- Find $\varrho =\varrho (v_{ij}{⇝}^{*}v_{pq})$
- $\forall p_{i}\in \varrho ,\forall \left(a,b\right)\in E=E\left(v_{ij}{⇝}_{\rho_{i}}v_{pq}\right),b\neq v_{pq},t_{ab}\neq -1$, Find the total time.
  (10) $$TotalTime_{\rho_{i}}=\left(\sum_{(a,b)\in E}t_{ab}\right)+\frac{D_{rv}}{S}+\frac{\Phi_{\hat{srv}}}{\omega }\mathrm{where}\;r\in L_{\rho_{i}}\left(v\right),s\in L_{\rho_{i}}\left(r\right)\mathrm{and}\;v=v_{pq}$$
- Pick $\rho_{k}\in \varrho$ such that
  (11) $$TotalTime_{\rho_{k}}=min_{\rho_{i}\in \varrho }\left\{TotalTime_{\rho_{i}}\right\}$$
- When reaching an edge with time cost t=−1, its value is replaced with the time cost obtained according to selected edges previously, as follows:
  (12) $$t_{rv_{pq}}=\frac{D_{\left(r,v\right)}}{S}+\frac{\Phi_{\hat{srv}}}{\omega }\mathrm{where}\;r,s,v\in V\left(v_{ij}{⇝}_{\rho_{k}}v_{pq}\right)\mathrm{and}\;v=v_{pq}$$
- The intermediate nodes on the selected path as well as $v_{pq}$ are added to χ. The path is built as we move from one edge to another.
- The edges that form the lowest cost are added to χ. The Algorithm 4 discards from I the intermediate nodes unselected neighbors and removes the edges with them.

The starting point key will define the sense of motion, for that the path will start in an upward or downward track (lines 1–4). For each brown node, the algorithm picks the next node according to the motion direction (lines 6–11). If this node is an adjacent node to the current one, the algorithm checks the key value for the next node. If the key of the next node is equal to 00 (lines 13–16), the edge between the current and next node is added to the set of edges χ (lines 34-35). In case the current or next node key is 11 (respectively 12) on the current track, then the next possible position on the upcoming track will be a node with either 00 or 12 (respectively 00 or 11) key-value (lines 17–19, respectively 20–23). To find the next possible position, we compare the possible paths between the two tracks (lines 25–27). These possible paths consist of edges between candidate turning points. The desired path is the one that has the lowest time cost and the highest coverage rate, that will be added to χ (line 28), and all the involved visited nodes are removed from the list of vertices I (line 29). Now that the turning point on the upcoming track is known, the algorithm checks if the node has an in-degree > 1 (which means that time cost $t_{pq}$ = −1). If so, the time cost for the edge can be computed using Equation (11) (line 30). When the path moves between two tracks the path direction is inversed (line 32). The Algorithm 4 repeats until no more vertices are found in I.
**Algorithm 4** Path Generation**Input:**$v_{ij}$: startpoint, G: Graph,**Output:**χ  1: upward=true
  2: **if** (key$\left(v_{ij}\right)$==11) **then**  3:     upward=false
  4: **end if**  5: **repeat**  6:     **if** ($v_{ij}$.color=brown) **then**  7:         **if** (upward==false) **then**  8:             $v_{next}$=$v_{i-1j}$  9:         **else**  10:             $v_{next}$=$v_{i+1j}$  11:         **end if**  12:         **if** ($v_{next}$ is an adjacent neighbor to $v_{ij}$) **then**  13:             **if** (key$(v_{next})=00$) **then**  14:                 $\chi =\chi \cup \{\left(v_{ij},v_{next}\right)\}$
  15:                 $I=I-\{v_{next}\}$
  16:                 $v_{{}^{\prime }}=v_{ij}$
  17:             **else**  18:                 **if** (key$(v_{next})=11$ key$(v_{ij})=11$ ) **then**  19:                   $k_{1}=00,k_{2}=12$
  20:                 **else**  21:                   **if** (key$(v_{next})=12$ key$(v_{ij})=12$) **then**  22:                       $k_{1}=00,k_{2}=11$  23:                    **end if**  24:                 **end if**  25:                 Find brown vertex $v_{pq}\in I$ key $\left(v_{pq}\right)=k_{1}$key$\left(v_{pq}\right)=k_{2}$ & $v_{ij}{⇝}^{*}v_{pq}$  26:                 **if** ($v_{pq}\neq$ null) **then**  27:                     Find $p_{k}\in \varrho \left(v_{ij}{⇝}^{*}v_{pq}\right)$ using Equations (10) and (11)  28:                     $\chi =\chi \cup E(v_{ij}{⇝}_{p_{k}}v_{pq})$  29:                     $I=I-V(v_{ij}{⇝}_{p_{i}}v_{pq})$  30:                     Assign time cost $t_{pq}$ using Equation (12)  31:                     $v_{{}^{\prime }}=v_{pq}$  32:                     upward=!upward  33:                 **else**  34:                     $\chi =\chi \cup \{\left(v_{ij},v_{next}\right)\}$  35:                     $I=I-\{v_{ij},v_{next}\}$  36:                 **end if**  37:             **end if**  38:             $v_{ij}=v_{{}^{\prime }}$  39:         **end if**  40:     **end if**  41:**until** (I=ϕ)

**Figure 10:**
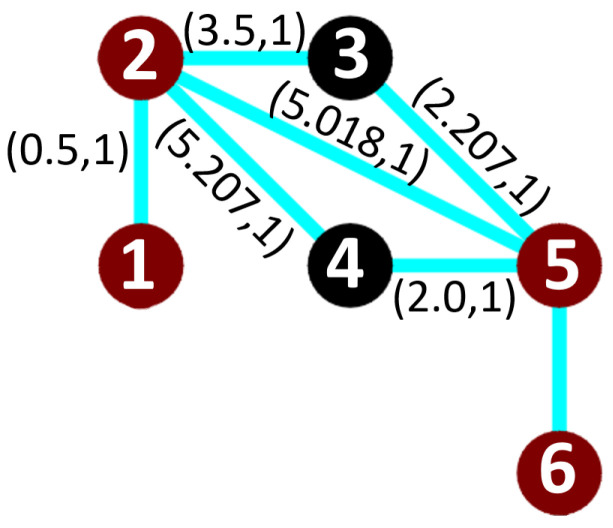
Turning node edges.

To examplify, Figure 10 shows the possible paths between turning node 2 with $deg^{+}\left(2\right)>1$, and the next possible position node 5, that has $deg^{-}\left(5\right)>1$, and $deg^{+}\left(5\right)==1$ with an unknown time cost ($t_{56}=-1$). Table 3 shows the possible time cost at edge (5,6). While Table 4 shows the list of possible paths from node 1 to node 6 and their time cost. As a result the best turning path is $\rho_{3}$ (1-2-5-6), and the weight of the edge(5,6) is 2.6 s

**Figure 11:**
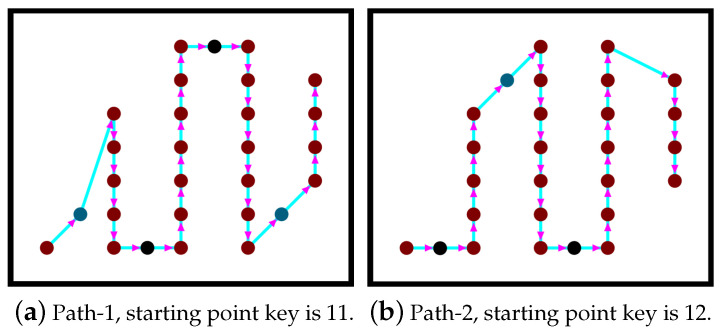
Two examples of generated paths.

As we have previously mentioned, there are two possible starting points, which are the first and the last brown points on the left column of the grid. The two starting points generate two possible paths (denoted path-1 and path-2). Our algorithm chooses the shortest path for the coverage mission. Figure 11 shows two different paths generated by our algorithm. The complexity of the path planning Algorithm 4 is o($V^{2}*E$). In the following section, we present our partitioning method called ”parallel partitioning along a side”, denoted PPS.

## 5. Partitioning Method (PPS)

The PPS method is applied when the area grid division phase terminates. It divides the area A by drawing lines (called partition borders), into *l* approximately equal sub-areas, denoted $A_{k}$ where k={1,…,l}. In each partition *k*, we generate a path $P_{k}$. In this section, we state and discuss the area partitioning method and the path planning phase using both cellular decomposition techniques: the exact and the approximate. The section is organized as follows. First, the area is partitioned using PPS-L (parallel partition along the length side), PPS-W (parallel partition along the width side), or PPS-T (parallel partition along both sides forming a T shape) (step 1). Then, we apply our border modification technique to refine the partition boundaries in the presence of NFZ (step 2). Finally, the path is planned using a single or multiple UAVs (step 3).

**Figure 12:**
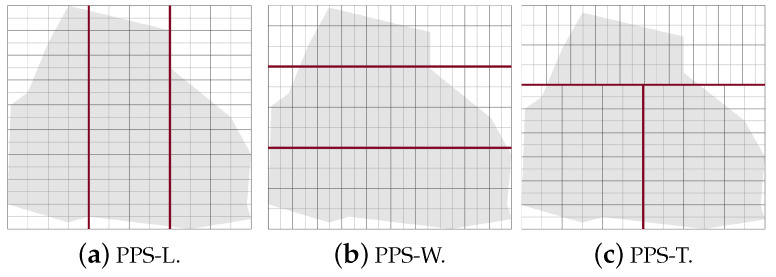
Examples of area partition using the three methods PPS-L, PPS-W and PPS-T.

Step 1: The partition borderlines are parallel to the shortest side of the grid cell. In other words, these borders are parallel to the longest side of the grid. If the longest side is the grid length, we call the partition method parallel partition along the length side (PPS-L) (Figure 12a). Otherwise, the partition borders are parallel to the width side of the grid, we call this method PPS-W (Figure 12b). In special cases, mainly in the presence of NFZ, the paths in the partitions may be interrupted. To prevent this from happening we either modify the partition borders (explained in step 2 below), or combine the two methods PPS-L and PPS-W to form a ”T shape partition” denoted PPS-T (Figure 12c). In PPS-T the two methods are applied one before the other according to the direction of the gird cell shortest side. The PPS-T method draws a horizontal line from the nearest row to the center of the NFZ, then it draws another line perpendicular to it to form the T-shape. The T-shape orientation could be horizontal (PPS-W then PPS-L) or vertical (PPS-L then PPS-W).

**Figure 13:**
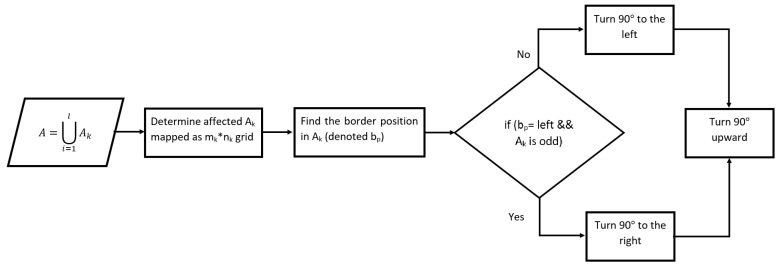
Modification border method flowchart.

Step 2: In some cases, the position of the NFZ interrupts the UAVs’ path that leaves portions of the area unreachable by one UAV and decreases the quality of coverage. In this case, we need to tune or modify the borderlines initially created. Figure 13 shows the workflow of the border modification method.

**Figure 14:**
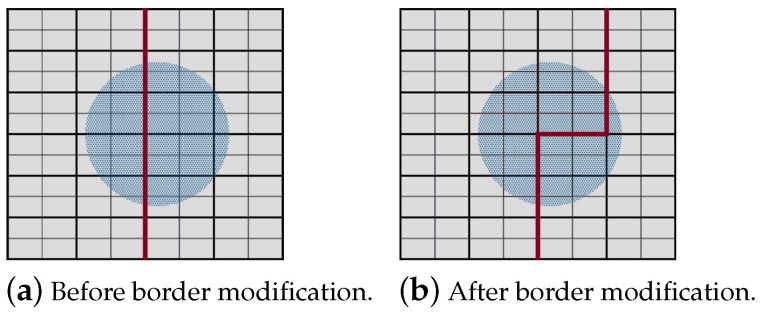
Border modification method sample.

We denote the partitioned sub-area $A_{k}$. For each partitioned $A_{k}$, the border to modify is the one that passes through the NFZ see Figure 14. This borderline is located on the right or left side of the sub-area. According to its location and the width of $A_{k}$ (i.e., number of columns in $A_{k}$), the line is turned 90° on the left or on the right, then another 90° upward if starting from the bottom side of the border. In case multiple UAVs are used, almost equal portions of the area are to be assigned to the UAVs, before the path planning process starts. For this goal, If PPS-L is applied and (m mod l) is greater than 1, we re-distribute the grid-columns among the partitions. The first (m mod l) partitions having the lowest number of vertices will have their number of columns increased by 1. The total number of columns becomes $\left(\frac{m}{l}+1\right)$ for these partitions. Similarly, if PPS-W is applied, we choose the first (n mod l) partitions, and we increase their number of rows by one $\left(\frac{n}{l}+1\right)$.

Step 3: In the coming subsections we present how our PPS method for planning the path works in case of exact cellular decomposition (Section 5.1), as well as approximate cellular decomposition in the presence of non-flying zones (Section 5.2). In case a single UAV is used, the planned paths are combined together for a total efficient path (Section 5.3).

### 5.1. PPS with Exact Cellular Decomposition

**Figure 15:**
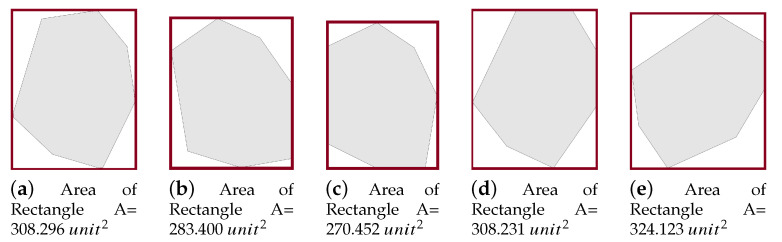
Possible generated rectangles that surrounds the area. The smallest rectangle is represented in (**c**).

**Figure 16:**
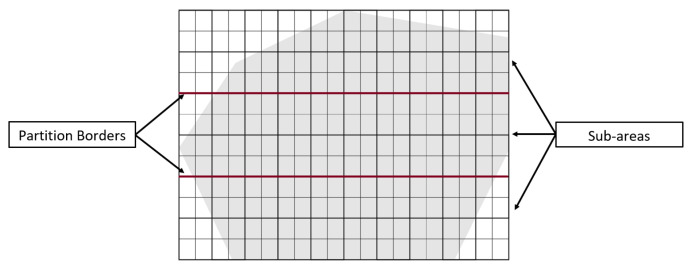
Area partitioned using PPS-W method.

To plan a path using exact cellular decomposition, we first find the smallest grid that can surround the area. For this, we choose the smallest rectangle (in terms of the area) around the edges of the area as shown in Figure 15. The reason is to select the smallest width side, which minimizes the number of columns and thus lowers the number of turns along the path. The chosen rectangle (Figure 15c) is then transformed into a grid and later PPS-W is applied as shown in Figure 16.

### 5.2. PPS with Approximate Cellular Decomposition

**Figure 17:**
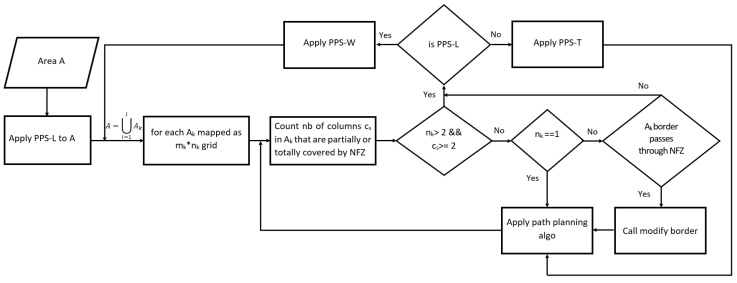
PPS flow chart in the presence of NFZ and using approximate cellular decomposition.

The position of the NFZ plays a major role in selecting the best partitioning direction. We assign a (−1) value to the cells that include parts of the NFZ. The planned path must not pass through the border lines. To cover areas in the presence of NFZ using approximate cellular decomposition, first the area must be partitioned and the path is planned on the obtained sub-areas [19,20]. The process starts by applying PPS-L partitioning, then checks the width of the sub-areas and the number of affected columns. According to these values, the PPS is changed to either PPS-W, PPS-T or a modification border is required. Figure 17 illustrates the work flow of the PPS method.

### 5.3. Combining Paths between Different Partitions

In case a single UAV covers the whole area of interest, the paths in the partitioned areas must be joined to form one complete path. The following rules allow the joining:

- We denote $v_{1}^{s_{i}}$ and $v_{1}^{e_{i}}$ (respectively $v_{2}^{s_{i}}$ and $v_{2}^{e_{i}}$) the start and end nodes of path-1 (respectively path-2) in partition $P_{i}$. In each partition $P_{i}$, these nodes are selected to form edges with the nearest boundary nodes denote $v^{b}$ (purple nodes). A connecting edge, denoted Γ, is an edge formed between two nodes $v\in G_{i}$ and $u\in G_{j}$, where $G_{i}$ and $G_{j}$ are the graphs representing two partitions $P_{i}$ and $P_{j}$, such that u.color=purple and $v\in \{v_{1}^{s_{i}},v_{1}^{e_{i}},v_{2}^{s_{i}},v_{2}^{e_{i}}\}$.
- We sort the edges cost for each partition according to the cost values, to choose the edges with minimum cost.
- If $P_{i}$ is connected to $P_{i+1}$, then nodes that belong to the connection edge between the partitions are not allowed to perform new connections.
- If a node in $P_{i}$ belongs to the connecting edge and it is a start node, then the second start node in the same partition (if exists) could not set connections with other partitions.
- If a node in $P_{i}$ belongs to the connecting edge and it is an end node, then the second end node in the same partition (if exist) could not set connections with other partitions.
- If $P_{i}$ is connected to $P_{i+1}$ and $P_{i+2}$, then $P_{i+1}$ and $P_{i+2}$ could not have a direct edge connection with each other.

**Figure 18:**
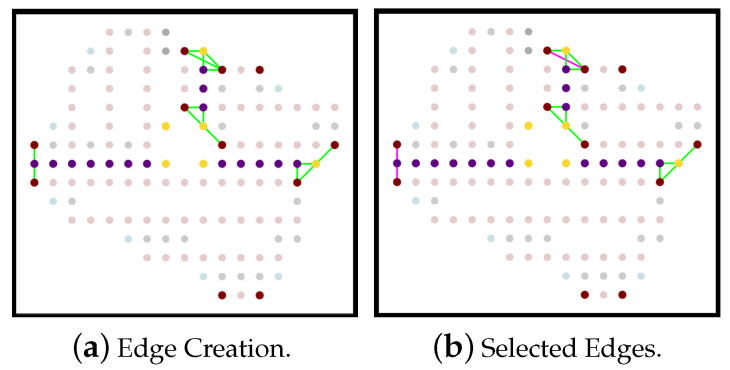
A sample of joining three partitioned areas.

Figure 18a shows the edges formed between partitions, and the pink colored edges, in Figure 18b, are the chosen edges. Below we present different possibilities for the joining phase:

- Alternative 1: in each partition area $P_{i}$, apply the joining rules on all start and end nodes in the partition paths.
- Alternative 2: in each partition area $P_{i}$, select the path-1. Then, select the start and end node for each partition path-1 and apply the joining rules.
- Alternative 3: in each partition area $P_{i}$, select the path-2. Then, select the start and end node for each partition path-2 and apply the joining rules.
- Alternative 4: in each partition area $P_{i}$, select the shortest path among path-1 and path-2. Then select the start and end node for each partition path and apply the joining rules.

**Figure 19:**
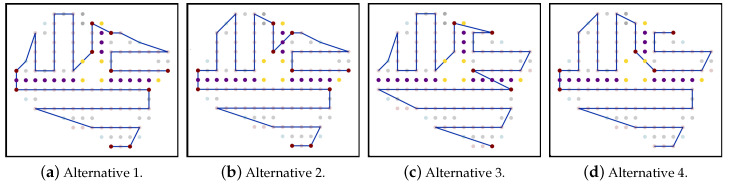
The paths generated by using each of the alternatives.

Successively, the algorithm will select the best path in terms of time and energy, as shown in Figure 19. In this example the path generated using total option 4 is the best total path.

## 6. Evaluation and Results

In this section, we show the comparative results of our work with different existing works. In the following, we first introduce the metrics used for the performance evaluation and then we show the scenarios we implemented and evaluated.

### 6.1. Evaluation Metrics

The performance is evaluated using the following metrics: energy consumption, completion time and quality of coverage.

- Total Completion Time: It is the total completion time for the planned path. It is computed by adding the time cost of all edges (u,v) on the path. We adopt the total completion time in [4] to evaluate our work.

(13) $$Total_{T}=\sum_{\left(u,v\right)}Time(u_{q},v_{p})$$

- Energy model: We adopt the energy model in [35]. Let W(u,v) denotes the energy cost of traversing an edge between nodes *u* and *v*.

(14) $$W\left(u,v\right)=\lambda D_{\left(u,v\right)}$$

where λ denotes the energy consumption per meter of length, $D_{\left(u,v\right)}$ is the distance. It is related to the UAV characteristics (speed, weight, power).

Let $W(\Phi_{\hat{uv^{{}^{\prime }}v}})$ be the cost associated with a feasible turn.

(15) $$W\left(\Phi_{\hat{uv^{{}^{\prime }}v}}\right)=\gamma \frac{180}{\pi }\Phi_{\hat{uv^{{}^{\prime }}v}}$$

where γ denotes the energy consumption per degree of turn angle. In our tests, we assume that *λ*= 0.1164 KJ/unit length and *γ*= 0.0173 KJ/degree.

The total energy *E* consumed is the sum of two weighted components. The first component is proportional to the distance traveled and the second is proportional to the sum of turning angles along the generated path.

(16) $$\Xi =\sum_{u\in V}\sum_{v\in V}W(u,v)+W\left(\Phi_{\hat{{\mathrm{uv}}^{{}^{\prime }}v}}\right)$$

- Quality of coverage: It computes the percentage of the area covered by the planned path. We adopt the quality of service formula in [19].

(17) $$QoC=\frac{CN}{TN}100\%$$

where CN is the number of non-zero cells that represent the area and covered by the planned path. TN is the number of non-zero cells in the area grid (excluding NFZ cells).

### 6.2. Results

In this section, we consider different scenarios and we compare our work to:

- The work in [30], where the area of interest is partitioned, and the path is planned using back-and-forth algorithm;
- The Bresenham’s line, which is used in [19,20];
- A grid-based algorithm in presence of NFZ, proposed in [16];
- The clustering method used to generate paths without collisions, presented in [34].

In all the scenarios, we assume that the UAVs are homogeneous. We assume that the UAVs travel with constant velocity, to be able to make a fair comparison among the different approaches. The use of constant velocity in calculations produces approximate results. In the scenarios, we used a velocity value that is equivalent to those used in [16,19,20,30,34]. The velocity value is considered as the average speed (less than the maximum speed) to achieve a fair comparison. It is important to note that the assumption for the UAVs to travel at a constant velocity could negatively affect our results. The paths planned by our approach consist of tracks that are parallel to the longest side in the grid. This generates long tracks where the UAVs could easily travel at a high speed for long distances. If we limit the speed at a specific level, this positive side of our work would be neglected. Furthermore, the total number of tracks (long and short tracks) in our path is less than the number of tracks in planned paths of other approaches. In the following, we discuss how the average speed could be computed, to understand which value could be considered as a fair value.

**Figure 20:**
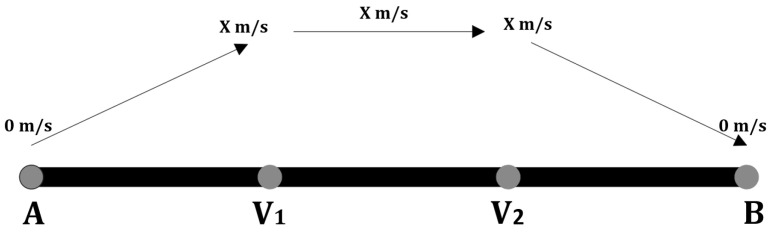
UAV horizontal speed variation while covering track AB.

For the sake of simplicity, let us assume that we want to cover a whole area by traveling at a constant velocity Y. Given a track AB that will be traversed by a UAV with horizontal speed between 0 and *X* m/s. In Figure 20, $AV_{1}$ represents the distance traversed with velocity between 0 and X m/s to reach the constant speed *X*. From $V_{1}$ to $V_{2}$ the UAV moves at a constant speed X m/s. Then from $V_{2}$ to *B*, the speed varies between *X* and 0 m/s. We choose *Y* to be the approximate average velocity (Y≤X m/s) to traverse the track AB, which enables a fair comparison to the different approaches. The value of *Y* is calculated as follows:

Let $t_{AB}$($S_{Y}$) be the time needed for the UAV to move from *A* to *B* with speed *Y* m/s, and $t_{AV_{1}}$($S_{0-X}$) be the time needed for the UAV to move from *A* to $V_{1}$ with speed variation between 0 and *X* m/s.

(18) $$t_{AB}\left(S_{Y}\right)=t_{AV_{1}}\left(S_{0-X}\right)+t_{V_{1}V_{2}}\left(S_{X}\right)+t_{V_{1}B}\left(S_{X-0}\right)$$

Since $t_{AV_{1}}\left(S_{0-X}\right)$ = $t_{V_{2}B}\left(S_{X-0}\right)$,

(19) $$t_{AB}\left(S_{Y}\right)=2*t_{AV_{1}}\left(S_{0-X}\right)+t_{V_{1}V_{2}}\left(S_{X}\right)$$

Consider that $t_{AV_{1}}\left(S_{0-X}\right)≃t_{AV_{1}}\left(S_{X/2}\right)$,

(20) $$t_{AB}\left(S_{Y}\right)=2*t_{AV_{1}}\left(S_{X/2}\right)+t_{V_{1}V_{2}}\left(S_{X}\right)$$

(21) $$\frac{d_{AB}}{Y}=2*\frac{d_{AV_{1}}}{\frac{X}{2}}+\frac{d_{V_{1}V_{2}}}{X}$$

If $d_{AV_{1}}$ = $d_{V_{1}V_{2}}$ = 1, then $d_{AB}$ =3. Thus,

$$\begin{array}{c} \frac{3}{Y}=\frac{2}{\frac{X}{2}}+\frac{1}{X}\\ \frac{3}{Y}=\frac{4}{X}+\frac{1}{X}\\ \frac{3}{Y}=\frac{5}{X}\\ Y=\frac{3}{5}X \end{array}$$

$d_{AV_{1}}\leq d_{V_{1}V_{2}}$, so to be fair we assume that $Y\leq \frac{3}{5}X$. In this work, the top speed *X* is 24 m/s, thus Y≤ 14.4 m/s. In the simulation we assume *Y* is equal to 10 m/s.

#### 6.2.1. Scenario 1

**Figure 21:**
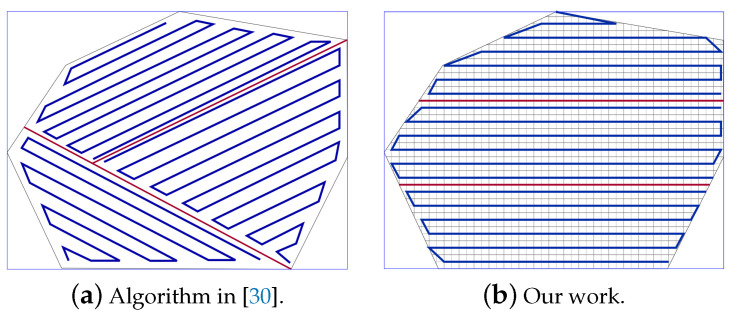
The paths generated by the algorithms in [30] and by our algorithm.

In this scenario, we compare our work to Maza et al. [30]. The area of interest is presented in Figure 21. The authors use the exact area of decomposition. They partitioned the area into three sub-areas and they use three UAVs for coverage. The planned path is generated using back-and-forth algorithm (Figure 21a). The path generated by our work is presented in Figure 21b. The PPS method is applied to select the smallest rectangle around the area. The UAVs speed is considered 8 unit/second with a rotation rate of 30 degrees/second.

Table 5 shows that the number of turning angles is decreased by 38.45% in our work. Both algorithms achieve the same QoC, which is 100%. However, the total path length in our work is 2.54% shorter than the path produced by [30], and still, the energy consumption is reduced by 5.02% and saves around 6.77% of completion time. To better understand the performance of our work and the gain in completion time. We varied both the speed (between 4 and 14 unit/second) and the rotation rate (between 10 and 60 degrees/second). Results show that the average completion time is 7.64% lower than [30].

#### 6.2.2. Scenario 2

In this scenario, we compare our PPS work to the algorithms provided by Valente et al. in [19,20]. They adopt an area of size approximately 327 m × 195 m. The UAVs speed is 10 m/second, and a rotation rate of 30 degree/second. We divide this scenario into three sub-scenarios and compare them according to the following: (A) We adopt the same partitioning method of [19,20], (B) we apply our PPS method along the length side, (C) we compare between the different solutions obtained by our PPS method (i.e., along the length side, along the width side and then “T-shape”).

**Figure 22:**
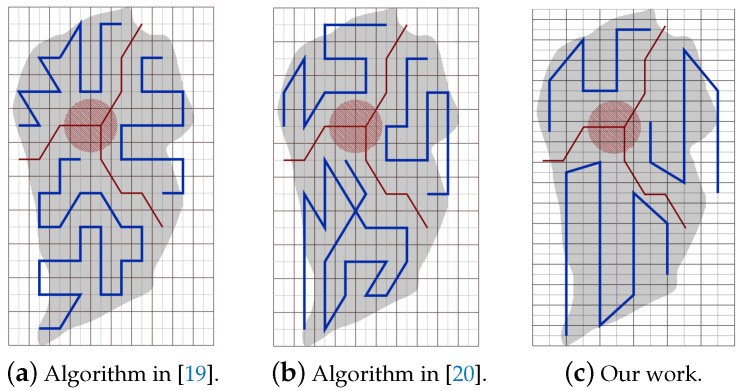
The paths generated by the algorithms in [19,20] and by our algorithm while using partion method in [19,20].

**Figure 23:**
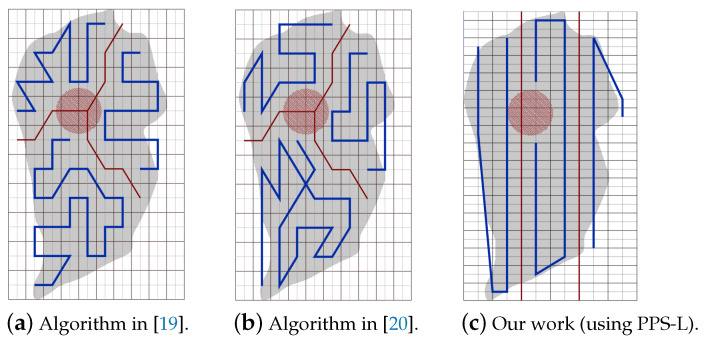
The paths generated by the algorithms in [19,20] and by our algorithm with PPS-L.

**Figure 24:**
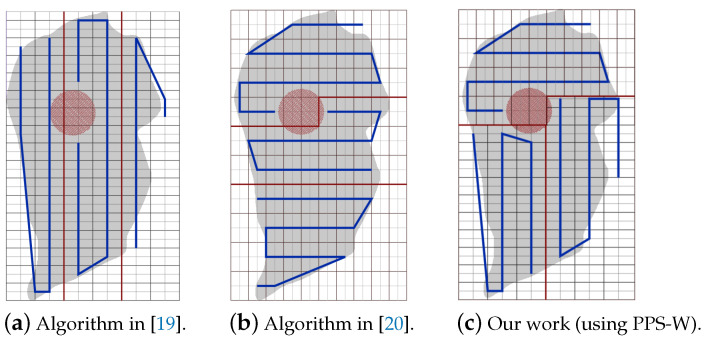
The paths generated by the algorithms in [19,20] and by our algorithm with PPS-W.

- Part A: Using the same partitioning method in [19,20]:
  Figure 22a,b represent the CPP done by [19,20], respectively. Both works exclude the border cells, which gives a quality of coverage QoC 72%. Figure 22c shows the CPP in our work.
  Table 6 shows that the total path’s length in our work is shorter than the paths in [19,20], by 8.02% and 20.18% respectively. In addition, our work lowers the total turning angles by 59.46% over [19] and by 51.03% over [30]. In our work, the completion time is reduced by 31.33% compared to [19], and 31.69% compared to [20]. The gain in energy is 21.90% in our work compared to [19], and 26.65% compared to [20]).
- Part B: PPS-L versus [19,20]
  We use our PPS-L method and we evaluate its performance. Figure 23a,b represent the CPP done by [19,20], respectively. Both works exclude the border cells, which gives a quality of coverage QoC of 72%. Figure 23c shows the CPP in our work and we can notice that achieves 100% of QoC.
  Table 7 shows that the total path length in our work is shorter by 3.70% than the paths generated by [20]. However, it is higher by 9.87% than the paths obtained by [19]. Our work lowers the total turning angles by 78.37% over [19] and by 73.88% over [20], respectively. Regarding the completion time, it is reduced by 29.52% compared to the time in [19]. It is also reduced by 29.89% compared to [20]. The gain in energy by PPS is 13.14% over [19] and 15.10% over [20].
- Part C: PPS-L versus PPS-W versus PPS-T
  In this section, we compare our 3 PPS methods on the area of [19,20] with same NFZ position, to evaluate the performance of each method. Figure 24a–c represents the CPP generated by our work using PPS-L, PPS-W and PPS-T, respectively.
  Table 8 shows that the total path length in our work using PPS-L is shorter than the paths using PPS-T by 7.72% and greater than PPS-W by 2.81%. PPS-L decreases the total turning angles by 50.00% over PPS-W and by 42.85% over PPS-T. In PPS-L, the completion time is reduced by 10.30% and 14.99% over PPS-W and PPS-T, respectively. The gain in energy is 4.09% and 12.85% over PPS-W and PPS-T, respectively.

#### 6.2.3. Scenario 3

In this scenario, the location of the NFZ determines which partition method to use. In this section, we study the performance of our partitioning methods by varying the position of the NFZ as follows: (a) NFZ located at the top side of the grid, (b) NFZ located at the right side of the grid and (c) NFZ located in the middle of the grid.

**Figure 25:**
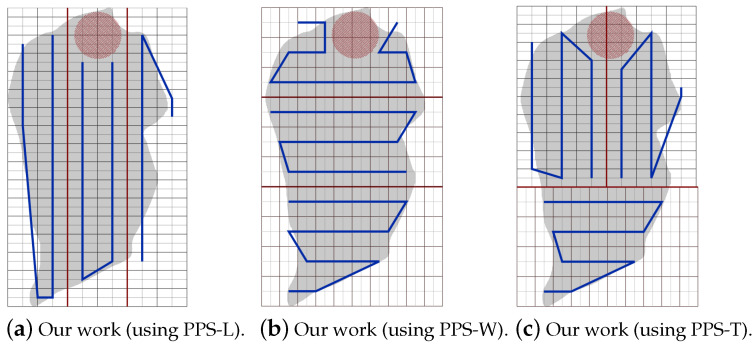
The paths generated by the algorithm by our algorithm using PPS-L, PPS-W and PPS-T with NFZ located at the top of the grid.

**Figure 26:**
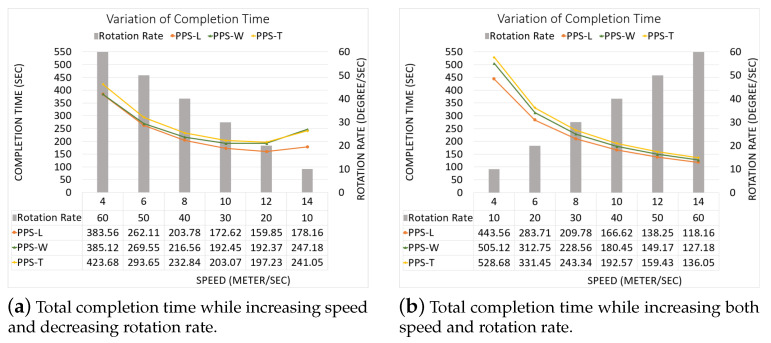
The variation of the total completion time in each area.

**Figure 27:**
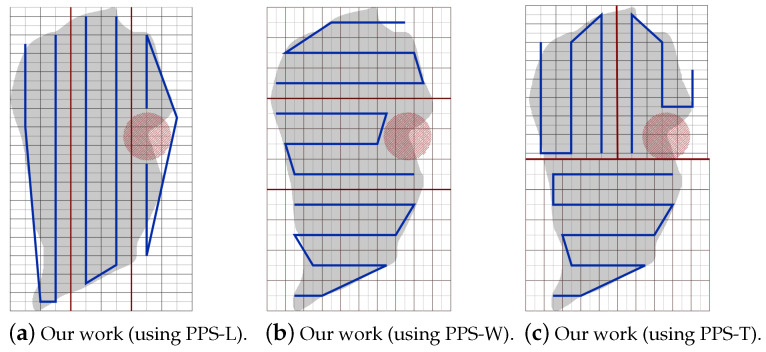
The paths generated by the algorithm by our algorithm using PPS-L, PPS-W and PPS-T with NFZ located at the right side of the grid.

**Figure 28:**
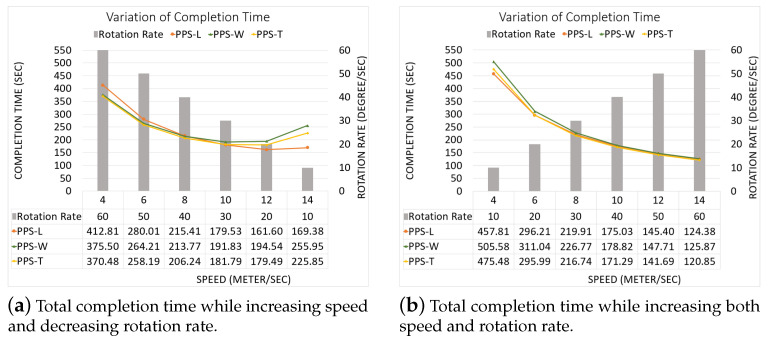
The variation of the total completion time in each area.

**Figure 29:**
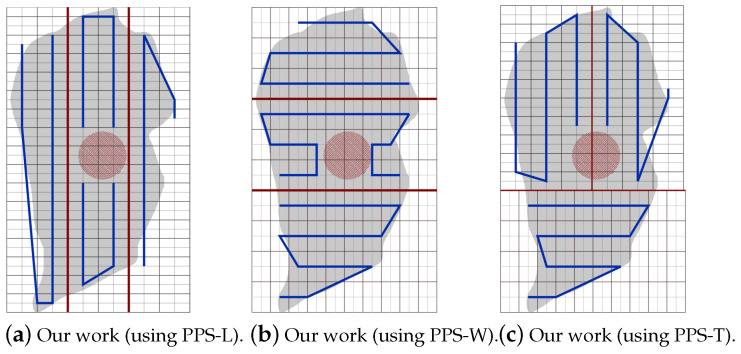
NFZ is located at the middle part of the grid.

**Figure 30:**
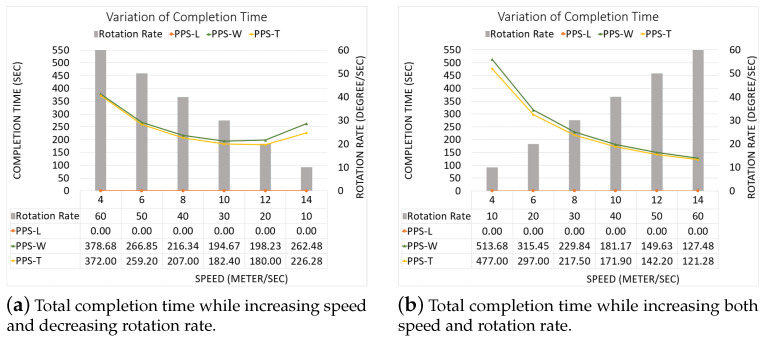
Gain in completion time in each sub-area.

- **NFZ located at the top side of the grid:**Figure 25a–c shows the CPP obtained by PPS-L, PPS-W and PPS-T, respectively.
  To evaluate the average performance for the partition methods, we assume that the speed is 9 m/s and rotation rate is 35 degree/sec. Table 9 shows that the PPS-W generated a shorter path than PPS-L and PPS-T by 2.15% and 2.43%, respectively, with lowering the turning angels in PPS-L by 65.41% and 57.14% than in PPS-W and PPS-T, respectively. The average completion time in PPS-L shows a gain by 12.84% and 10.74% against PPS-W and PPS-T, respectively, with lowering the energy by 7.39% and 6.84% against PPS-W and PPS-T, respectively.
  Figure 26 shows the variation of the total completion time in each area. The results are obtained while increasing both speed and rotation rate (Figure 26a), and also while decreasing speed with the increase of the rotation rate (Figure 26b).
- **NFZ located at the right side of the grid:**Figure 27a–c shows the CPP obtained by PPS-L, PPS-W, PPS-T, respectively.
  To evaluate the average performance for the partitioning methods, we consider the speed of 9 m/s and rotation rate equal to 35 degree/s. Table 10 shows that PPS-W generates a shorter path than PPS-L and PPS-T. The path is shorter in PPS-W by 13.45% and 4.29%, respectively. PPS-L decreases the turning angles by 65.40% and by 57.14% over PPS-W and PPS-T, respectively. On the other hand, the average completion time in PPS-W shows a gain of 4.37% and 5.95% over PPS-L and PPS-T, respectively. PPS-W also lowers the energy consumption by 7.39% and 5.34% over PPS-L and PPS-T, respectively.
  Figure 28 shows the variation of the completion time in each area. In Figure 28a, the results are obtained by increasing both speed and rotation rate. In Figure 28b, we decrease speed while increasing the rotation rate.
- **NFZ located in the middle of the grid:** The CPP by PPS-L, PPS-W and PPS-T, are presented in Figure 29a–c, respectively. The PPS-L method was unable to plan the path in subarea 2 since the NFZ covers cells in two columns of the area which divides it into two disjoint areas. To evaluate the average performance of the partitioning methods, we fix the speed to 9 m/s with rotation rate 35 degree/sec.
  Table 11 shows that PPS-T generates a shorter path than PPS-W with a slight difference of 0.20%. It lowers the turning angles in PPS-T by 22.22% over PPS-W. The average completion time in PPS-T is reduced by 5.22% over PPS-W, with energy saving of 3.53%.
  The results shown in Figure 30 present the gain in completion time in each area. The results in Figure 30a are obtained while increasing both speed and rotation rate. Figure 30b shows the completion time while decreasing speed and increasing the rotation rate.

#### 6.2.4. Scenario 4

**Figure 31:**
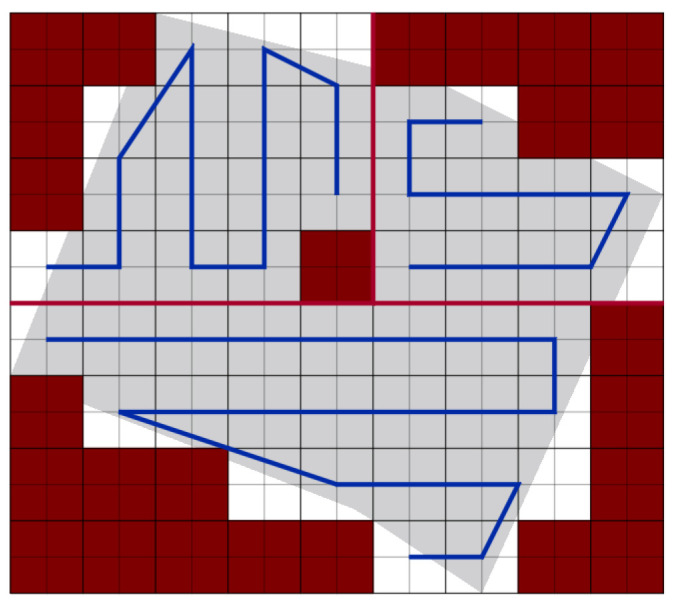
Planned path after partition.

In this scenario, we compare our path planning algorithm to the algorithm provided in Di Franco et al. [16]. Authors in [16] plan the path in the presence of NFZ. They use two minimum-cost methods (O-F) and (E-F). The O-F method calculates the lower cost paths based on the sum of the total turning angles, while the E-F computes the energy consumption estimation. In our work, we partition the area using our PPS method, then we plan the path, then the partition borders are removed and the paths are connected to cover the area using a single UAV. Figure 31 shows the area after applying PPS-T method. Note that the NFZ cells are the red cells.

**Figure 32:**
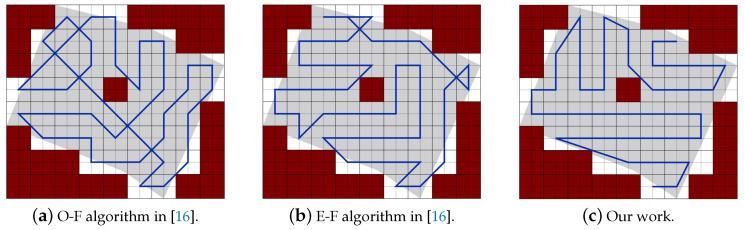
Paths generated by the two algorithms in [16] and by our algorithm.

**Figure 33:**
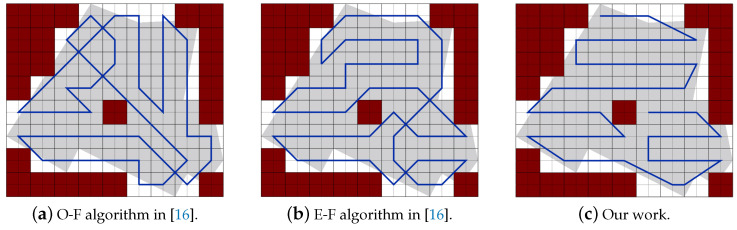
Paths generated by the two algorithms in [16] and by our algorithm.

Area 1: Figure 32 represents the CPP generated in the work of [16] using O-F and E-F methods, and the obtained CPP in our work.
  The results in Table 12 shows that the total paths length of our work is shorter than the paths of [16] using O-F and E-F methods by 16.10% and 8.62% respectively, and lowering the summation of the turning angles by 20.00% and 21.57% respectively. In our work the completion time is reduced by 18.34% and 16.38% than paths provided by O-F and E-F, respectively. It also achieves a gain of 17.56% and 13.80% in the consumed energy against paths planned by O-F and E-F respectively.
Area 2: Figure 33 represents the CPP generated in the works of [16] using O-F and E-F methods, and the obtained CPP in our work.
  The results in Table 13 show that the total path’s length of our work is shorter than the paths of [16] using O-F and E-F methods by 12.60% and 10.04%, respectively. PPS method lowers the total turning angles by 17.33% in both. In our work the completion time is reduced by 15.14% and 14.01% over O-F and E-F methods, respectively. It achieves a gain of 14.21% and 12.58% in the consumed energy against paths planned by O-F and E-F, respectively.

#### 6.2.5. Scenario 5

**Figure 34:**
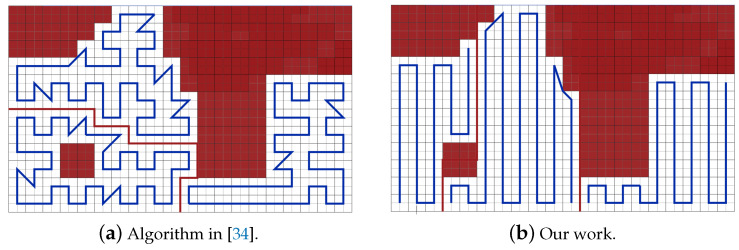
Paths generated by the algorithms in [34] and by our algorithm.

In this scenario, we compare our path planning algorithm to the algorithm provided in [34] work. Authors in [34] plan the path in the presence of NFZ. Figure 34 represents the CPP generated in the works in [34] and the obtained CPP in our work.

The results in Table 14 show that the total path length of our work is shorter than the paths of [34] by 3.72%, and lowering the sum of the turning angles by 68.60%. In our work, the completion time is reduced by 48.96%. Our work also achieves a gain of 36.59% in the consumed energy against the work in [34].

## 7. Conclusions

In this paper, we proposed an energy-aware path planning algorithm to cover an area of interest in the presence of NFZ, using a novel parallel partition method. The proposed solution can be applied in scenarios where a single and multiple UAVs are used. The area of interest is divided into a grid using a grid-based technique with subdivision. The area is also partitioned into several sub-areas according to the number of UAVs. In case a single UAV is used single UAVs a path joining algorithm is provided, to connect the paths over the sub-areas taking into account the energy, time and coverage metrics. The area partitioning algorithm divides the area into approximately equal sub-areas, in a way that the partition borders pass over the cells borders to avoid dropping those cells during the path planning phase. The performance of our solution is comparatively evaluated using three metrics: completion time, energy consumption and quality of coverage. In the future works, we aim at enhancing the joining path algorithm, and evaluating the work in the presence of multiple obstacles/NFZ of different shapes and sizes. 

## Figures and Tables

**Table 1:** Comparison table of our approach over related works in this paper.

		Our Work	[16]	[19]	[20]	[30]	[34]
**Area Cellular decomposition**	Approximate	*	*	*	*		*
	Exact	*				*	
**Area decomposition Technique**	Grid-based	*	*	*	*		*
	Grid Sub-division	*					
**Non-flying zones**	Presence of NFZ inside the area	*	*	*	*		*
	NFZ located around the area	*	*				*
	More than one NFZ inside the area						*
**Area Partitioning**	Provide Partition algorithm	*		*	*	*	*
	Exclude partition border cells			*	*		
**Number of UAVs**	Single UAV	*	*				
	Multiple UAVs	*		*	*	*	*
**Grid to Graph mapping**	Graph representation	*	*	*	*		*
	Graph filtering	*					
**Edges Weighting**	Provide cost function for edges	*	*	*	*		
	Include coverage ratio in the weight	*					
**Evaluation Metrics**	Energy consumption	*	*				*
	Completion time	*	*	*	*	*	*
	Quality of Coverage	*		*	*		

* The features of each work are indicated using the star symbol.

**Table 2:** Table of Notations.

Notation	Description	Notation	Description
**A**	Geographical area	**$t_{uv}$**	Time needed to move between two vertices
**$A_{k}$**	Sub-area in A	**$c_{uv}$**	Coverage value for an edge
**$v_{ib_{j}}$**	vertex $v_{i}$($x_{i}$,$y_{i}$) located on the column $b_{j}$	***D***	Distance between two nodes *u* and *v*
**$b_{j}$**	Grid column	**S**	UAV speed
**$G_{k}$**	Formed graph	**ω**	UAV rotation rate
**U**	Set of UAVs	**L(u)**	Set of all predecessors between two vertices
**P**	UAV Path	**$\rho_{i}$**	Set of possible simple paths
**T**	Set of turning points	**$E(u{⇝}_{\rho_{i}}v)$**	Set of edges along a path $\rho_{i}$
**Φ**	Turning angles	**$L_{\rho_{i}}\left(v\right)$**	Direct predecessor of vertex v along the path $\rho_{i}$
**$P_{total}$**	Total path	**$L_{\rho_{i}}^{s}\left(u\right)$**	Direct successor to vertex u along the path $\rho_{i}$
**G(V,E,ζ)**	Grid represented in Graph	$\varrho (u{⇝}^{*}v)$	All the possible paths between *u* and *v*
**V**	Set of vertices/nodes	**I**	List of vertices
**E**	Set of edges	**χ**	Set of edges that form the lowest cost
**ζ**	Set of colors	***l***	Number of sub areas in A
**β**	Edge label	***m***	The total number of columns
**α**	Vertices label	***n***	The total number of rows
**K**	Set of keys	**$v_{1}^{s_{i}}$**	Start node of path
**$C_{p}^{q}$**	Sub-column p in major column q	**$v_{1}^{e_{i}}$**	End node of path
**$R_{i}^{j}$**	range i of vertices in column j	**Γ**	Connecting edge between two graphs
**$R_{i}^{j}.min$**	Set the min bound for Range i	**W(u,v)**	Energy cost of traversing an edge
**$R_{i}^{j}.max$**	Set the max bound for Range i	**$W(\Phi_{\hat{uv^{{}^{\prime }}v}})$**	Energy cost associated with a feasible turn
**$F_{1}$**	Row of first vertex in $C_{p}^{q}$	**λ**	Energy consumption per meter of length
**$L_{1}$**	Row of last vertex in $C_{p}^{q}$	**γ**	Energy consumption per degree of turn angle
**deg(v)**	Degree (or valency) of a vertex *v*	**Ξ**	Total energy consumed
**$deg^{-}\left(v\right)$**	Indegree of a vertex *v*	**CN**	Number of covered non-zero cells
**$deg^{+}\left(v\right)$**	Outdegree of a vertex *v*	**TN**	Number of the non-zero cells in the area grid

**Table 3:** Possible time cost for edge(5,6).

	From	To	$\Phi_{\hat{s56}}$	$t_{rv_{56}}$
**1**	edge(2,5)	edge(5,6)	63	2.6
**2**	edge(3,5)	edge(5,6)	45	2
**3**	edge(4,5)	edge(5,6)	90	3.5

**Table 4:** Possible paths from node 1 to node 6.

	$\varrho (1{⇝}^{*}2)$	${\mathrm{TotalTime}}_{\rho_{i}}$
**$\rho_{1}$**	1-2-3-5-6	8.207
**$\rho_{2}$**	1-2-4-5-6	11.207
**$\rho_{3}$**	1-2-5-6	8.118

**Table 5:** Coverage path planning (CPP) path provided in Ref. [30] vs. our CPP using our PPS-L partition method.

	(a) Ref. [30]	(b) Our Work
**Route length [unit] ⊤**	9422.20	9182.56
**UAVs speed [unit/s] υ**	8	8
**Turns [degree]**	4680	2880
**Rotation rate [deg/sec]** **ω**	30	30
**Completion-time [sec]** **τ**	1333.77	1243.45
**Energy consumption [KJ] $\Xi_{total}$**	1177.71	1118.48

**Table 6:** CPP path provided in Ref. [30] and Ref. [20] vs. our CPP using same partition method.

	(a) Ref. [19]	(b) Ref. [20]	(c) Our Work
**Route length ⊤ in meters**	1339.52	1543.46	1231.97
**UAVs speed [m/s] υ**	10	10	10
**Turns [degree]**	3330	2757	1350
**Rotation rate [deg/sec]** **ω**	30	30	30
**Completion-time [sec]** **τ**	244.95	246.24	168.19
**Energy consumption [KJ] $\Xi_{total}$**	213.53	227.35	166.75

**Table 7:** CPP path provided in Ref. [30] and Ref. [20] vs. our CPP using our PPS-L partition method.

	(a) Ref. [19]	(b) Ref. [20]	(c) PPS-L
**Route length ⊤ in meters**	1339.52	1543.46	1486.23
**UAVs speed [m/s] υ**	10	10	10
**Turns [degree]**	3330	2757	720
**Rotation rate [deg/sec]** **ω**	30	30	30
**Completion-time** **τ** **in s**	244.95	246.24	172.623
**Energy consumption [KJ] $\Xi_{total}$**	213.53	227.35	185.45

**Table 8:** Our CPP using our PPS-L partition method vs. PPS-W partition method vs. PPS-T partition method.

	(a) PPS-L	(b) PPS-W	(c) PPS-T
**Route length ⊤ in meters**	1486.23	1444.48	1610.71
**UAVs speed [m/s] υ**	10	10	10
**Turns [degree]**	720	1440	1260
**Rotation rate [deg/sec]** **ω**	30	30	30
**Completion-time [sec]** **τ**	172.62	192.44	203.07
**Energy consumption [KJ] $\Xi_{total}$**	185.45	193.05	209.28

**Table 9:** Our CPP using our PPS-L partition method vs. PPS-W partition method vs. PPS-T partition method.

	(a) PPS-L	(b) PPS-W	(c) PPS-T
**Route length ⊤ in meters**	1433.19	1402.29	1437.25
**UAVs speed [m/s] υ**	9	9	9
**Turns [degree]**	540	1561	1260
**Rotation rate [deg/sec]** **ω**	35	35	35
**Completion-time [sec]** **τ**	174.67	200.41	195.69
**Energy consumption [KJ] $\Xi_{total}$**	176.17	190.23	189.09

**Table 10:** Our CPP using our PPS-L partition method vs. PPS-W partition method vs. PPS-T partition method.

	(a) PPS-L	(b) PPS-W	(c) PPS-T
**Route length ⊤ in meters**	1615.25	1397.92	1460.65
**UAVs speed [m/s] υ**	9	9	9
**Turns [degree]**	540	1561	1260
**Rotation rate [deg/sec]** **ω**	35	35	35
**Completion-time [sec]** **τ**	200.07	191.32	203.44
**Energy consumption [KJ] $\Xi_{total}$**	200.49	184.52	194.93

**Table 11:** Our CPP using our PPS-L partition method vs. PPS-W partition method vs. PPS-T partition method.

	(a) PPS-L	(b) PPS-W	(c) PPS-T
**Route length ⊤ in meters**	N/A	1406.70	1403.98
**UAVs speed [m/s] υ**	9	9	9
**Turns [degree]**	N/A	1620	1260
**Rotation rate [deg/sec]** **ω**	35	35	35
**Completion-time [sec]** **τ**	N/A	202.58	191.99
**Energy consumption [KJ] $\Xi_{total}$**	N/A	185.07	178.53

**Table 12:** CPP path provided in Ref. [16] vs. our CPP.

	(a) O-F	(b) E-F	(c) Our Work
**Route length ⊤ in meters**	556.98	511.42	467.33
**UAVs speed [m/s] υ**	10	10	10
**Turns [degree]**	2250	2295	1800
**Rotation rate [deg/sec]** **ω**	30	30	30
**Completion-time [sec]** **τ**	130.69	127.64	106.73
**Energy consumption [KJ] $\Xi_{total}$**	103.76	99.23	85.54

**Table 13:** CPP path provided in Ref. [16] vs. our CPP.

	(a) O-F	(b) E-F	(c) Our Work
**Route length ⊤ in meters**	582.84	566.27	509.38
**UAVs speed [m/s] υ**	10	10	10
**Turns [degree]**	2025	2025	1674
**Rotation rate [deg/sec]** **ω**	30	30	30
**Completion-time [sec]** **τ**	125.78	124.12	106.74
**Energy consumption [KJ] $\Xi_{total}$**	102.87	100.94	88.25

**Table 14:** CPP path provided in Ref. [34] vs. our CPP.

	(a) Ref. [34]	(b) PPS-L
**Route length ⊤ in meters**	1575.56	1517.02
**UAVs speed [m/s] υ**	10	10
**Turns [degree]**	10,890	3420
**Rotation rate [deg/sec]** **ω**	30	30
**Completion-time [sec]** **τ**	520.56	265.70
**Energy consumption [KJ] $\Xi_{total}$**	371.79	235.75
